# A225 VACCINE-PREVENTABLE DISEASES IN HOSPITALIZED PATIENTS WITH END-STAGE LIVER DISEASE/CIRRHOSIS: A NATIONWIDE COHORT ANALYSIS

**DOI:** 10.1093/jcag/gwab049.224

**Published:** 2022-02-21

**Authors:** D Hudson, R Khanna, M Brahmania, K Qumosani, A Teriaky

**Affiliations:** Department of Gastroenterology, Western University, London, ON, Canada

## Abstract

**Background:**

Cirrhosis is associated with immune dysfunction, which increases susceptibility to infection and subsequent hospitalization. Vaccination of this high-risk patient population can mitigate the risk of infection.

**Aims:**

Data from the National Inpatient Sample (NIS) was analyzed to compare the prevalence of vaccine-preventable diseases (VPD) among hospitalized patients both with and without cirrhosis.

**Methods:**

The 2013 NIS database was interrogated using ICD-9-CM codes to identify patients with cirrhosis and VPD. Baseline characteristics were compared (see: **Table 1**). Univariate and multivariate regression models identified risks associated with VPD adjusting for survey procedures.

**Results:**

313,710 patients were hospitalized for VPD, including 13,080 patients (4.1%) with cirrhosis (see: **Table 1**) Patients with cirrhosis were more likely to be hospitalized with pneumococcal pneumonia (odds ratio [OR] = 1.45 [95% CI 1.29 – 1.63], P <0.001), hepatitis A (OR = 7.04 [95% CI 5.96 – 8.31], *P <0.001*) and hepatitis B (OR = 14.41 [95% CI 12.53 – 14.36], *P <0.001*) infections compared to patients without liver cirrhosis. Patients with cirrhosis were less likely to have an infection with influenza (OR = 0.55 [95% CI 0.49 – 0.62], *P <0.001*), human papillomavirus (HPV) (OR = 0.57 [95% CI 0.43 – 0.75, *P < 0.001*) and varicella zoster (OR = 0.78 [95% CI 0.69 – 0.89], *P <0.001*). Minimal differences in hospitalizations for haemophilus influenzae or meningococcal infections were noted between groups.

Odds ratios for VPD adjusting for age, sex, race, patient location, patient income, hospital type and bed-size, mortality risk, type 2 diabetes mellitus, malignancy, human immunodeficiency virus (HIV), organ transplantation and immunodeficiency:

pneumococcal pneumonia (OR = 1.27 [95% CI 1.13 – 1.44], *P < 0.001*), hepatitis A (OR = 5.99 [95% CI 5.02 – 7.15], *P < 0.001*); and hepatitis B (OR = 11.07 [95% CI 10.24 – 11.97], *P < 0.001*).

**Conclusions:**

These results emphasize the importance of vaccinating patients with cirrhosis against pneumococcal pneumonia, hepatitis A and hepatitis B infections to reduce hospitalization

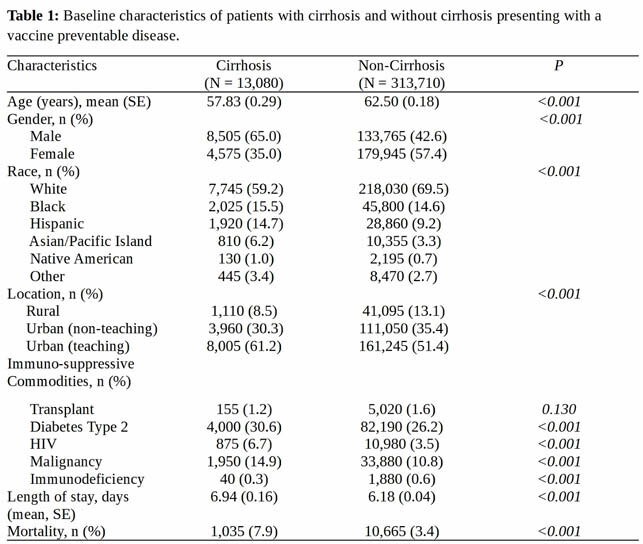

**Table 1:** Baseline characteristics of patients with cirrhosis and without cirrhosis presenting with a vaccine preventable disease.

**Funding Agencies:**

None

